# Nine-Month Trend of IgG Antibody Persistence and Associated Symptoms Post-SARS-CoV-2 Infection

**DOI:** 10.3390/healthcare12090948

**Published:** 2024-05-05

**Authors:** Angel Lugo-Trampe, Daniel López-Cifuentes, Paúl Mendoza-Pérez, Yaliana Tafurt-Cardona, Alejandra de Jesús Joo-Domínguez, Clara Patricia Rios-Ibarra, Marisol Espinoza-Ruiz, Consuelo Chang-Rueda, Iram Pablo Rodriguez-Sanchez, Margarita L. Martinez-Fierro, Iván Delgado-Enciso, Karina del Carmen Trujillo-Murillo

**Affiliations:** 1 Faculty of Human Medicine, Campus IV, Universidad Autónoma de Chiapas, Tapachula 30700, Mexico; 2 Genodiagnóstica SA de CV, Tapachula, Chiapas 30700, Mexico; 3 Faculty of Chemistry Sciences, Campus IV, Universidad Autónoma de Chiapas, Tapachula 30700, Mexico; 4 Medical and Pharmaceutical Biotechnology Unit, Center for Research and Assistance in Technology and Design of the State of Jalisco (CIATEJ), Guadalajara 44270, Mexico; 5 Molecular and Structural Physiology Laboratory, School of Biological Sciences, Universidad Autónoma de Nuevo León, San Nicolás de los Garza 66455, Mexico; 6 Molecular Medicine Laboratory, Unidad de Medicina Humana y Ciencias de la Salud, Universidad Autónoma de Zacatecas, Zacatecas 98160, Mexico; 7 School of Medicine, University of Colima, Colima 28040, Mexico; 8 Colima Cancerology State Institute, IMSS-Bienestar, Colima 28085, Mexico

**Keywords:** COVID-19 serological testing, SARS-CoV-2 infection antibody testing, seroconversion, COVID-19 reinfection, antibody responses

## Abstract

Between 2 and 8.5% of patients who recover from COVID-19 do not develop antibodies, and the durability of IgG antibodies is under scrutiny. Therefore, the presence and persistence of IgM and IgG antibodies were evaluated in a group of patients diagnosed with SARS-CoV-2 from May to August 2020. Out of 2199 suspected COVID-19 cases, 1264 were confirmed for SARS-CoV-2 by rRT-PCR; 328 consented to participate in the study, with 220 participants followed for 9 months, including 124 men (56%) and 96 women (44%). The primary symptoms were headache, dry cough, and fever. IgG antibodies developed in 95% of patients within 4 weeks post-diagnosis, and a second evaluation at 9 months showed that 72.7% still had detectable IgG antibodies. The presence of IgM in one individual (0.45%) suggested the possibility of reinfection.

## 1. Introduction

In late December 2019, a novel zoonotic pathogen known as severe acute respiratory syndrome coronavirus 2 (SARS-CoV-2) emerged, leading to the outbreak of the infectious respiratory condition named coronavirus disease 2019 (COVID-19). Distinct from previous coronaviruses such as SARS-CoV-1 and MERS-CoV, SARS-CoV-2 has had an unprecedented spread globally with increased morbidity [1].

As reported by the World Health Organization (WHO), by December 2023, there had been approximately 773.8 million confirmed cases and 7.01 million deaths attributable to COVID-19 globally [2]. In Mexico, there were 7.5 million reported cases and about 333,913 deaths due to the disease as of May 2023. Specifically, in the state of Chiapas, Mexico, there were 58,428 cases and 2253 deaths [3], while the city of Tapachula confirmed 9239 cases [4].

SARS-CoV-2 gains entry into cells by binding to angiotensin-converting enzyme 2 (ACE2) and utilizing transmembrane serine protease 2 (TMPRSS2) for S protein priming. This serine protease facilitates the cleavage of the S protein into S1 and S2 subunits, enabling the fusion of the viral and cellular membranes [5,6].

The virus is composed primarily of four structural proteins: the spike (S), membrane (M), envelope (E), and nucleocapsid (N) proteins [7]. The N protein, which is vital for the viral life cycle, contains three conserved domains: the N-terminal domain (NTD), C-terminal domain (CTD), and the N3 region [8], which plays a crucial role in viral replication, assembly, and immune regulation [9].

COVID-19 presents with a wide spectrum of clinical manifestations, ranging from fever, cough, and fatigue, which are the most common symptoms, to nasal congestion, runny nose, and diarrhea in a small portion of patients. It can vary from asymptomatic and mild symptoms to severe conditions such as interstitial pneumonia, acute respiratory distress syndrome (ARDS), septic shock, and difficult-to-treat metabolic acidosis, as well as hemorrhagic and coagulation dysfunction [10]. The variability in disease expression among patients is influenced by factors including age, sex, and comorbidities such as cancer, diabetes, hypertension, obesity, and cardiovascular diseases [11]. A multivariate analysis conducted by Jang et al. in 2023 indicated that PCR positivity for COVID-19 was associated with a longer stay in the intensive care unit (ICU), increasing the risk of morbidity and mortality associated with the severity of the infection [12].

Upon viral entry, the host immune system can recognize the virus or its epitopes, triggering an innate or adaptive immune response [13]. Typically, infections with SARS-CoV-2 lead to the production of IgM and IgG antibodies [14,15]. Currently, it is understood that serum viral antibodies undergo a slight increase in the early phase of the disease. Subsequently, patients with COVID-19 exhibit a gradual rise in virus-specific IgG and IgM levels up to the third week after symptom onset, after which IgM levels begin to decline while IgG levels continue to rise, maintaining stable IgG titers for approximately three months. IgM levels are more indicative of recent infection during the early phase of the disease, whereas IgG responses become more predictive of infection from day 8 onwards after symptom onset [16]. The concentration of antibodies in patients’ serum has demonstrated a negative correlation with viral RNA levels, indicating an effort to neutralize and eliminate the virus [17,18].

The study of IgG antibody persistence in the context of COVID-19 is crucial for understanding the potential for long-term immunity against the virus, informing vaccination strategies, and assessing the risk of reinfection. Unlike the transient nature of IgM, IgG antibodies signify a more lasting immune response, pivotal for the neutralization and memory of viral infections. This becomes particularly significant when comparing the persistence of antibodies in COVID-19 to other coronaviruses like SARS-CoV and MERS-CoV, for which antibody responses have been noted to last from several months to a few years, highlighting potential parallels and distinctions in immune durability [19,20]. Such comparisons provide invaluable insights into the expected longevity of protective immunity and underscore the importance of monitoring antibody levels post-recovery from COVID-19.

Interestingly, higher antibody titers observed in severely ill patients suggest a correlation with disease severity, whereas a weaker antibody response may be associated with more efficient viral clearance [21]. Between 2 and 8.5% of patients who recover from COVID-19 do not develop antibodies, and the durability of IgG antibodies is under scrutiny [22,23]. According to Zhao et al. (2020), antibody detection holds significant diagnostic value as it provides clinical evidence during the course of SARS-CoV-2 infection, supporting the routine application of serological assays [24].

This study aims to detail the symptoms, comorbidities, and the durability of the antibody response to SARS-CoV-2 in the population of Tapachula, Chiapas, Mexico—a region bordering Guatemala.

## 2. Materials and Methods

This study employed an observational, prospective, longitudinal design to gather data on antibody detection against SARS-CoV-2. Conducted from May to August 2020 at the Genodiagnóstica Laboratory, which is situated in Tapachula, Chiapas, Mexico, the researchers utilized a facility certified by the Institute for Epidemiological Diagnosis and Reference (InDRE) ‘Dr. Manuel Martínez Báez’ for SARS-CoV-2 identification (Official Document Number: DGE-DDYR-DSAT-03558-2020).

Patients were eligible for inclusion in the study if they met the operational definition of a suspected case of viral respiratory disease according to the Ministry of Health guidelines and were confirmed for SARS-CoV-2 infection via the gold standard, a real-time reverse transcription–polymerase chain reaction (rRT-PCR) test. The criteria aimed to encompass a broad spectrum of the population affected by COVID-19, including various ages, genders, and clinical severities. Exclusion criteria included individuals who were unable to provide informed consent or who had a history of travel outside of the region within 14 days prior to symptom onset, to reduce the risk of including imported cases which might not reflect local transmission dynamics. Upon enrollment, clinical and demographic data were captured using the Ministry of Health’s case study format for viral respiratory diseases. Blood samples were collected at Genodiagnóstica Laboratory using Gold Top BD Vacutainer™ Venous Blood Collection Tubes: SST Serum Separation Tubes at two time points: 23–28 days and 9 months after the initial positive confirmation of SARS-CoV-2 infection by rRT-PCR. These samples were analyzed to evaluate the presence of SARS-CoV-2 IgM and IgG antibodies.

The time frames of 23–28 days and 9 months post-initial positive rRT-PCR confirmation were strategically chosen based on current scientific understanding of the immune response to SARS-CoV-2 infection. The early testing window (23–28 days) was selected to assess the initial development of IgM and IgG antibodies, coinciding with the period when antibody responses are expected to peak following acute infection. The 9-month marker was chosen to evaluate the persistence of IgG antibodies, providing insights into the longevity of the immune response and potential for lasting immunity. These time points are critical for understanding both the short-term and long-term dynamics of antibody production in response to SARS-CoV-2 infection.

### 2.1. Ethical Considerations

The study protocol was evaluated and approved by the Institutional Review Boards of the General Direction for Research and Postgraduate Studies (registration number: 03/MHT/RPR/222/20) and the Research Ethics Committee of the Faculty of Human Medicine ‘Dr. Manuel Velasco Suárez’, Campus IV, at the Autonomous University of Chiapas, in accordance with the Declaration of Helsinki. Participation was voluntary, with patient consent being documented through signed informed consent forms. In cases in which subjects were unable to sign, consent was obtained from a legal representative.

### 2.2. Procedures

The methodology procedure for detecting and analyzing SARS-CoV-2 encompassed several key stages: 1. Collection of pharyngeal and nasopharyngeal samples; 2. Blood collection for serum analysis; 3. Viral isolation using the QIAamp Viral RNA Kit (QIAGEN, P/N 52906, Hilden, Germany); 4. Identification of SARS-CoV-2 from clinical samples by rRT-PCR using the SuperScript™ III Platinum™ One-Step qRT-PCR Kit (Thermo Fisher Scientific, P/N 11732088, Carlsbad, CA, USA); 5. Molecular diagnosis of coronavirus SARS-CoV-2 using the 2019-nCoV Kit from Integrated DNA Technologies (IDT) ( Coralville, IA, 10006606, USA), in accordance with the protocols established by the Centers for Disease Control and Prevention (CDC); and 6. Detection of IgM and IgG antibodies against 2019-nCoV using the Maglumi 2019-nCoV IgM/IgG chemiluminescence assay in serum samples from SNIBE Diagnostics, Shenzhen, China.

### 2.3. rRT-PCR for SARS-CoV-2

The isolation of viral RNA was conducted using the QIAamp^®^ Viral RNA Mini Kit (QIAGEN, P/N 52906, Hilden, Germany) with 140 µL of UTM viral transport medium (COPAN Diagnostics Inc., Murrieta, CA, USA) for pharyngeal and nasopharyngeal exudate samples, according to the manufacturer’s instructions. The isolated RNAs were subjected to rRT-PCR testing for the SARS-CoV-2 virus, using 5 µL of RNA for identification with the SuperScript™ III Platinum™ One-Step qRT-PCR Kit. The reaction mixture, with a total volume of 25 µL, included ultrapure water, 2X reaction buffer mix, MgSO4 (50 mM), and primers (forward and reverse, both at 10 µM), as well as probes (10 µM), following the CDC 2019-nCoV Real-Time RT-PCR Diagnostic Panel guidelines for SARS-CoV-2 detection. In each run, a blank (NTC), positive control (C+), extraction reagent control (CRE), and negative control (C-) were included, along with the amplification of the human RNase P (RP) gene as an endogenous quality control for the presence of human RNA.

The identification of the SARS-CoV-2 virus targeted the conserved regions of the nucleocapsid gene (N1, N2, and N3) using the following specific primers and probes: 2019-nCoV_N1-F 5′-GACCCCAAAATCAGCGAAAT-3′, 2019-nCoV-N1-R 5′-TCTGGTTACTGCCAGTTGAATCTG-3′, 2019-nCoV_N1-P 5′-FAM-ACCCCGCATTACGTTTGGTGGACC-BHQ1-3′; 2019-nCoV_N2-F 5′-TTACAAACATTGGCCGCAAA-3′, 2019-nCoV-N2-R 5′-GCGCGACATTCCGAAGAA-3′, 2019-nCoV_N2-P 5′-FAM-ACAATTTGCCCCCAGCGCTTCAG-BHQ1-3′; 2019-nCoV_N3-F 5′-GGGAGCCTTGAATACACCAAAA-3′, 2019-nCoV-N3-R 5′-TGTAGCACGATTGCAGCATTG-3′, 2019-nCoV_N3-P 5′-FAM-AYCACATTGGCACCCGCAATCCTG-BHQ1-3′; and the amplification of the internal control RP RP-F 5′-AGATTTGGACCTGCGAGCG-3′, RP-R 5′-GAGCGGCTGTCTCCACAAGT-3′, RP-P 5′-FAM–TTCTGACCTGAAGGCTCTGCGCG–BHQ-1-3′, described in the IDT 2019-nCoV protocol. The CDC-established amplification conditions included reverse transcription for 35 min at 50 °C, RT denaturation/DNA polymerase activation for 5 min at 95 °C, and 45 PCR cycles of 10 s at 95 °C and 35 s at 55 °C. The rRT-PCR results are reported as cycle-threshold (Ct) values, which are inversely proportional to the initial amount of RNA in the sample. Ct values ≤ 40 were clinically reported as positive for SARS-CoV-2 in the PCR testing of the samples.

### 2.4. Detection of SARS-CoV-2 Antibodies

Between 23 and 28 days and 9 months after the positive confirmation of SARS-CoV-2 in patients by rRT-PCR, a comprehensive analysis was conducted to detect IgM and IgG antibodies specific to SARS-CoV-2. This detection was carried out using the Maglumi 2019-nCoV IgM/IgG chemiluminescence assay, a state-of-the-art diagnostic tool specifically designed for qualitative analysis in serum samples on a Snibe MAGLUMI 2000 Chemiluminescence Immunoassay (CLIA) system. The assay utilizes magnetic microbeads coated with recombinant antigens from the nucleocapsid and spike proteins of 2019-nCoV, which serve as the foundation for capturing SARS-CoV-2-specific antibodies present in the serum samples of patients. Additionally, the assay incorporates anti-human IgM and IgG antibodies labeled with ABEI (*N*-(aminobutyl)-*N*-(ethylisoluminol)), allowing for the precise detection of IgM and IgG antibodies directed against SARS-CoV-2 antigens. The serum sample volume analyzed for each patient was 10 µL, ensuring optimal levels of sensitivity and accuracy in antibody detection. The results, expressed as relative light units (RLUs), are proportional to the concentration of IgM and IgG antibodies to SARS-CoV-2 in each patient’s serum sample. According to the manufacturer’s instructions, a result <1.00 AU/mL is considered non-reactive, whereas a result ≥1.00 AU/mL is considered reactive, suggesting the presence of IgM and/or IgG antibodies specific to SARS-CoV-2.

### 2.5. Statistical Analysis

Descriptive statistics for categorical variables were presented as absolute frequencies and percentages. For continuous variables, they were described using the mean, standard deviation, and median along with the interquartile range age variable. Data were analyzed using chi-square (X^2^) test to identify statistically significant differences, with a *p* value ≤0.05 indicating statistical significance. The statistical analyses were performed using SPSS version 25.0 (SPSS Inc., Chicago, IL, USA) software.

## 3. Results

Among 2199 suspected cases of COVID-19 analyzed between May and August of 2020, 1264 were confirmed positive for SARS-CoV-2 by rRT-PCR. Of these patients, 328 consented to participate in this study (Table 1). The proportion of men (67%, n = 220) was higher than that of women (33%, n = 108). The most affected age range for both genders was 31 to 60 years, with a mean age of 43.73 ± 8.25 years. Table 1 details the clinical characteristics of these patients. The most frequent symptoms for men included fever (72.5%), cough (66.7%), headache (62.7%), myalgia (56.9%), and arthralgia (56.9%), while for women, the most common symptoms were fever (60%), cough (76%), headache (80%), myalgia (64%), and general state affliction (68%).

The study observed notable differences in antibody persistence when the participants were categorized into three age groups: ≤30, 31–60, and ≥61 years. The persistence of IgG antibodies at 9 months post-infection was highest among the 31–60 age group, with a retention rate of 75%, compared to 68% in the ≤30 group and 65% in the ≥61 group. This trend suggests a more robust long-term antibody response in middle-aged individuals, which may reflect that optimal immune maturation has not yet been achieved in younger individuals or it is possibly declining in older individuals. Symptom severity also varied across age groups, with the ≥61 age group experiencing a higher incidence of severe symptoms, including dyspnoea and ARDS, underscoring the impact of aging on disease outcomes.

**Figure 1:**
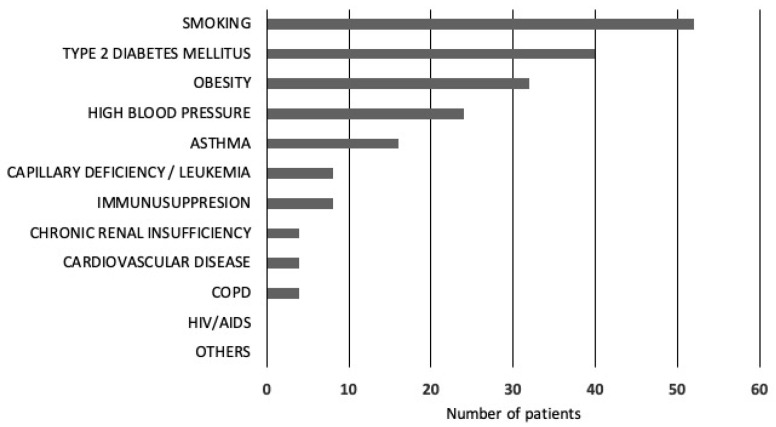
Comorbidities in 120 asymptomatic COVID-19 patients.

Of the 304 symptomatic patients, 184 had no comorbidities. The remaining 120 presented with one or more of the following conditions: smoking (n = 52, 43.3%), type 2 diabetes mellitus (T2DM) (n = 40, 33.3%), obesity (n = 32, 26.6%), hypertension (n = 24, 20.0%), asthma (n = 16, 13.3%), capillary deficiency or leukemia (n = 8, 6.6%), immunosuppression (n = 8, 6.6%), renal insufficiency (n = 4, 3.3%), chronic obstructive pulmonary disease (COPD) (n = 4, 3.3%), and cardiovascular disease (n = 4, 3.3%) (Figure 1).

### 3.1. First Detection of Antibodies (IgM and IgG)

Between 23 and 28 days after receiving SARS-CoV-2-positive diagnoses, the detection of IgM and IgG antibodies against the virus was performed for 316 patients (Table 2). Positivity for IgM was identified in 108 men and 52 women (OR = 1.63, 95% CI = 0.7304–1.8521, *p* = 0.524), while positivity for IgG was identified in 192 men and 100 women (OR = 0.96, 95% CI = 0.3972–2.3201, *p* = 0.927).

### 3.2. Second Detection of Antibodies (IgM and IgG)

Nine months after a positive COVID-19 diagnosis was confirmed by rRT-PCR, follow-up testing for IgM and IgG antibodies was conducted in 220 patients (Table 3); IgM antibodies were detected in only one male patient. IgG antibodies remained detectable in 101 men and 59 women (OR = 3.423, 95% CI = 1.8316–6.3997, *p* = 0.0001).

## 4. Discussion

In the study group, 328 positive cases for SARS-CoV-2 were detected by rRT-PCR, of which 24 patients were asymptomatic, and 304 were symptomatic. The percentage of men was higher (67%, n = 220) than that of women (33%, n = 108). The most affected age group for both genders was 31 to 60 years (43.73 ± 8.25 years). These results align with an epidemiological analysis by the Hospital of Guangzhou Medical University (China, 2020), which studied 1590 confirmed cases and found a mean age of 48 years, with 51.9% being male patients [25]. Such findings suggests that geographic, genetic, and lifestyle factors may influence COVID-19 outcomes, reinforcing the WHO’s recommendation for age-based prognoses [2].

The clinical characteristics of confirmed cases revealed that the most frequent symptoms in men were fever (72.5%), cough (66.7%), headache (62.7%), myalgia (56.9%), and arthralgia (56.9%); women most commonly reported fever (60%), cough (76%), headache (80%), myalgia (64%), and general state affliction (68%). N. Chen et al. (2020) also identified fever (87.9%), dry cough (67.7%), lymphopenia (82.1%), dyspnea, and pneumonia in severe cases, while diarrhea was less common [26].

Within the symptomatic subgroup, 184 patients had no comorbidities, whereas 120 had at least one, predominantly T2DM (33.3%,), hypertension (20.0%), obesity (26.6%), renal failure (3.3%), immunosuppression (6.6%), COPD (3.3%), cardiovascular disease (3.3%), and smoking (43.3%). Notably, the incidence of T2DM was higher in men (35.3%) compared to women (16.0%), and a greater proportion of women had multiple comorbidities (12% vs. 9.8% in men).

The comorbidities observed in this study are consistent with those reported in the literature, with diabetes and hypertension being prominent [27]. A meta-analysis has suggested a significant presence of these comorbidities, along with heart disease and obesity, in patients with MERS-CoV [28]. Age and comorbidities are well established as risk factors for severe complications of COVID-19 [29]. Conditions like T2DM and chronic respiratory disease may compromise the immune system, affecting the host’s innate immune response [30].

The prevalence of antibodies against SARS-CoV-2 is aligned with the existing literature, suggesting a high seroprevalence of IgG antibodies shortly after infection [31]. However, our findings indicate that the presence of IgG does not differ significantly between men and women (192 and 100, respectively; OR = 0.96, 95% CI = 0.3972–2.3201, *p* = 0.927) [32], echoing reports that do not show sex-based differences in the seroprevalence of SARS-CoV-2 and other coronaviruses [33,34].

An analysis by gender revealed distinct patterns in both antibody persistence and symptom severity. Out of the 220 participants followed for IgG antibody persistence, 101 men still had detectable IgG antibodies at the 9-month follow-up, compared to 59 women. However, the severity of symptoms and the rate of hospitalization demonstrated clear gender differences. Men reported severe symptoms more frequently and were hospitalized at a higher rate than women. This observation is consistent with broader trends noted in COVID-19 research, suggesting that gender may play a role in the disease’s impact, potentially due to biological factors such as differences in immune response, as well as socio-behavioral factors that influence health outcomes.

A small subset of patients might not seroconvert within 23–28 days post-infection, potentially due to T-cell-mediated immunity or localized mucosal immune response that do not elicit systemic IgG antibodies [23,35]. This could be attributed to a rapid cellular immune response to SARS-CoV-2 infection that may control the virus below the threshold required to trigger a measurable humoral response [36]. Meanwhile, the literature on other coronaviruses, such as SARS-CoV or MERS-CoV, indicates that antibodies can persist for about one to two years [37,38]. SARS-CoV-2 appears to elicit a more transient IgG response, with antibody levels diminishing 2–3 months post-infection [39].

Zhou W. et al. (2020) noted a decrease in neutralizing antibodies in some COVID-19 convalescent patients around 6–7 weeks after the onset of the disease, further complicating our understanding of long-term immunity [40].

Our study’s observations regarding the prevalence of symptoms and comorbidities among males could be attributed to lower levels of androgens in women potentially conferring a protective effect against COVID-19 [41]. Smoking and T2DM were particularly notable comorbidities within the study population; smoking has been associated with increased COVID-19 severity (OR ranging from 3 to 12) [42], and T2DM has been shown to double the mortality risk compared to that of non-diabetic patients [43].

At 9 months post-diagnosis, our analysis revealed that 27.3% of patients (60 out of 220 tested) had undetectable IgG antibodies against SARS-CoV-2, raising concerns about the longevity of humoral immunity post-infection [40]. In Tapachula, Chiapas, symptomatic individuals displayed persistent detectable IgG anti-SARS-CoV-2 antibodies (72.7%) for at least 9 months post-infection. This correlation between clinical manifestations and antibody persistence could inform future preventive and therapeutic strategies for COVID-19.

The persistence of IgG antibodies 9 months post-infection underscores the potential for long-term immunity against SARS-CoV-2, a finding that aligns with a recent longitudinal study that found that IgG antibodies against the spike protein of SARS-CoV-2 remained detectable in a majority of patients for up to 8 months post-infection [44]. However, our observation of IgG persistence contrasts with earlier concerns of rapidly waning immunity, as reported by some studies in the initial months of the pandemic. These discrepancies could be attributed to differences in study populations, methodologies for antibody detection, or even the viral strains involved. Notably, there are a lack of studies supporting the sustained presence of IgM antibodies against SARS-CoV-2, suggesting that the detection of IgM in one patient at nine months might indicate a reinfection rather than a prolonged IgM response. This highlights the need for further investigation into the mechanisms underlying the persistence and functionality of IgM in COVID-19 recovery.

The study’s methodology had several limitations that could impact the interpretation of our results. Firstly, the reliance on participant self-reporting for initial symptom onset dates may introduce recall bias, affecting the accuracy of the timing for antibody testing. Secondly, the study did not account for asymptomatic individuals or those with mild symptoms who did not seek testing, potentially skewing the data towards more severe cases. Additionally, variations in individual immune responses, influenced by factors such as age, sex, and pre-existing conditions, could not be fully controlled in the study design.

Conducting this study amidst a global pandemic presented unique challenges, particularly in terms of participant recruitment and follow-up. Restrictions on movement, healthcare system overload, and the participants’ fear of exposure to SARS-CoV-2 at medical facilities could deter participation and impact follow-up rates. To mitigate these challenges, the study employed a combination of remote consent procedures, when necessary, and ensured that all in-person interactions adhered strictly to safety protocols recommended by health authorities. Moreover, maintaining communication with participants for the 9-month follow-up required dedicated effort, particularly as the pandemic evolved and individuals’ circumstances changed.

These methodological considerations and challenges highlight the complexities of conducting longitudinal serological studies during a pandemic and underscore the importance of flexible study designs to adapt to unforeseen obstacles.

## 5. Conclusions

Our study provides critical insights into the persistence of IgG antibodies up to nine months following SARS-CoV-2 infection, highlighting a significant potential for long-term immunity. The detection of IgG antibodies in 72.7% of participants at the nine-month follow-up suggests a durable immune response in a substantial proportion of recovered individuals. This enduring response, however, contrasts with earlier concerns about rapidly waning immunity, underscoring the variability of immune persistence observed in different studies, possibly due to differences in methodologies, population demographics, and viral strains.

Notably, the rare detection of IgM antibodies at this late stage in one individual hints at the complexity of the immune response, potentially indicating a case of reinfection rather than prolonged IgM persistence. This observation aligns with the lack of literature supporting sustained IgM responses, suggesting that such occurrences are atypical and warrant further investigation to understand the mechanisms of reinfection and immune memory in the context of COVID-19.

Furthermore, the absence of detectable antibodies in some individuals could be influenced by rapid cellular immune responses that might control the infection before a significant humoral response is required. This aspect highlights the need for comprehensive studies that include cellular immunity assessments to fully elucidate the protective impacts of immune responses post-infection.

Overall, our findings contribute to the growing body of knowledge on COVID-19, providing valuable data for shaping future research, informing public health strategies, and guiding vaccination policies to combat the ongoing pandemic effectively.

## Figures and Tables

**Table 1:** Clinical parameters by gender for patients infected with SARS-CoV-2.

	Patients	Men	Women	Mean ± SD	Min/Max
**Age**	n = 328	220 (67%)	108 (33%)	42.14 ± 11.94	21/74
≤30	60 (18.3%)	32 (14.5%)	28 (26%)	26.0 ± 2.86	21/30
31–60	252 (76.8%)	176 (80%)	76 (70.3%)	43.73 ± 8.25	31/60
≥61	16 (4.8%)	12 (5.5%)	4 (3.7%)	68.66 ± 3.55	64/74
**Clinical manifestations**	304 (94%)	204 (92.7%)	100 (92.6%)		
**Signs and symptoms on admission**				**OR (95% CI), *p***	**X^2^, *p***
Fever	208 (68.4%)	148 (72.5%)	60 (60%)	1.64 (1.02–2.63), 0.039	9.308, 0.002 *
Cough	212 (69.7%)	136 (66.7%)	76 (76%)	0.68 (0.41–1.11), 0.128	4.245, 0.039 *
Headache	208 (68.4%)	128 (62.7%)	80 (80%)	0.48 (0.29–0.80), 0.005 *	2.769, 0.096
Dyspnoea	100 (32.9%)	64 (31.4%)	36 (36%)	0.82 (0.50–1.34), 0.433	1.960, 0.162
Irritability	116 (38.2%)	64 (31.4%)	52 (52%)	0.44 (0.27–0.71), 0.0008 *	0.310, 0.577
Diarrhea	144 (47.4%)	88 (43.1%)	56 (56%)	0.61 (0.38–0.98), 0.042 *	1.778, 0.182
Chest pain	104 (34.2%)	68 (33.3%)	36 (36%)	0.89 (0.54–1-46), 0.657	1.385, 0.239
Shivers	120 (39.5%)	72 (36%)	48 (48%)	0.60 (0.37–0.97), 0.039 *	1.200, 0.273
Odynophagia	108 (35.6%)	56 (27.5%)	52 (52%)	0.36 (0.22–0.59), 0.0001*	0.037, 0.847
Myalgia	180 (59.2%)	116 (56.9%)	64 (64%)	0.76 (0.48–1.22), 0.264	3.756, 0.053
Arthralgia	164 (54%)	116 (56.9%)	48 (48%)	1.39 (0.87–2.21), 0.159	7.049, 0.008 *
General attack	168 (55.3%)	100 (49%)	68 (68%)	0.49 (0.30–0.78), 0.003 *	1.524, 0.217
Rhinorrhea	104 (34.2%)	60 (29.4%)	44 (44%)	0.54 (0.33–0.88), 0.014 *	0.615, 0.433
Polypnea	68 (22.4%)	44 (21.6%)	24 (24%)	0.87 (0.49–1.53), 0.64	1.471, 0.225
Vomiting	28 (9.2%)	20 (9.8%)	8 (8%)	1.25 (0.53–2.93), 0.60	1.286, 0.257
Abdominal pain	44 (11.5%)	24 (11.8%)	20 (20%)	0.53 (0.28–1.02), 0.060	0.091, 0.763
Conjunctivitis	8 (2.6%)	0	8 (8%)	0.02 (0.00–0.46), 0.013 *	NA
Cyanosis	4 (1.3%)	4 (2%)	0	4.51 (0.24–84.54), 0.313	NA
Anosmia	44 (14.55)	28 (13.7%)	16 (16%)	0.83 (0.43–1.62), 0.602	0.818, 0.366
Dysgeusia	40 (13.2%)	28 (13.7%)	12 (12%)	1.16 (0.56–2.39), 0.674	1.600, 0.206
**Comorbidities**					
None	184 (60.5%)	112 (54.9%)	72 (72%)	0.51 (0.32–0.83), 0.007 *	2.174, 0.140
At least one	88 (28.9%)	72 (35.3%)	16 (16%)	2.79 (1.53–5.10), 0.0008 *	8.909, 0.003 *
More than two	32 (10.5%)	20 (9.8%)	12 (12%)	0.80 (0.37–1.70), 0.562	0.500, 0.480

The *p* values represent the comparison between the values observed by gender. * Value below the cutoff point. SD: Standard deviation. Bold is to highlight values with statistical significance.

**Table 2:** Detection of IgM and IgG antibodies for SARS-CoV-2 after 23–28 days post-diagnosis by rRT PCR.

IgM against SARS-CoV-2					IgG against SARS-CoV-2				
	Men	Women	OR	*p* Value		Men	Women	OR	*p* Value
**Positive**	108	52	1.163	0.524	**Positive**	192	100	0.96	0.927
**Negative**	100	56			**Negative**	16	8		
**Total**	208	108			**Total**	208	108		

A value of *p* ≤ 0.05 reflects statistically significant differences. OR between genders were estimated for IgM and IgG.

**Table 3:** Detection of IgM and IgG antibodies for SARS-CoV-2, 9 months post-diagnosis by rRT-PCR.

IgM against SARS-CoV-2					IgG against SARS-CoV-2				
	Men	Women	OR	*p* Value		Men	Women	OR	*p* Value
**Positive**	1	0	2.47	0.57	**Positive**	101	59	3.423	0.0001 *
**Negative**	120	99			**Negative**	20	40		
**Total**	121	99			**Total**	121	99		

* A value of *p* ≤ 0.05 reflects statistically significant differences. OR between genders were estimated for IgM and IgG.

## Data Availability

Data are unavailable due to privacy or ethical restrictions.
